# Assessment of Red Blood Cell Distribution Width-to-Platelet Count Ratio as a Prognostic Indicator for Acute Pancreatitis Severity and Outcomes: A Prospective Observational Study

**DOI:** 10.7759/cureus.97618

**Published:** 2025-11-23

**Authors:** Vemula Raja Babu, Ravikiran H R, Kalyani Raju, Prabhavathi K, Anil K Sakalecha

**Affiliations:** 1 General Surgery, Sri Devaraj Urs Medical College, Sri Devaraj Urs Academy of Higher Education and Research (SDUAHER), Kolar, IND; 2 Pathology, Sri Devaraj Urs Medical College, Sri Devaraj Urs Academy of Higher Education and Research (SDUAHER), Kolar, IND; 3 Biochemistry, Sri Devaraj Urs Medical College, Sri Devaraj Urs Academy of Higher Education and Research (SDUAHER), Kolar, IND; 4 Radiology, Sri Devaraj Urs Medical College, Sri Devaraj Urs Academy of Higher Education and Research (SDUAHER), Kolar, IND

**Keywords:** acute pancreatitis, disease severity, icu stay, inflammatory marker, mortality, platelet count, prognosis, red cell distribution width (rdw)- to- platelet ratio, roc curve, rpr

## Abstract

Background

Acute pancreatitis (AP) presents with a wide range of severity, from mild to life-threatening disease. Early identification of severe cases is crucial. Traditional prognostic scores such as Ranson's Criteria, Acute Physiology and Chronic Health Evaluation II (APACHE II), and Bedside Index of Severity in Acute Pancreatitis (BISAP) are accurate but complex. The red cell distribution width-to-platelet ratio (RPR), derived from a routine complete blood count (CBC), has emerged as a simple, inexpensive alternative.

Methods

A prospective observational study was conducted at R.L. Jalappa Hospital and Research Centre, Kolar, from June to August 2025, including 58 adult patients with acute pancreatitis (AP). Red cell distribution width (RDW) and platelet counts were obtained at admission, 48 hours, and 72 hours to calculate the red cell distribution width-to-platelet ratio (RPR). Outcomes, including severity, organ failure, intensive care unit (ICU) stay, and mortality, were analyzed and compared with conventional prognostic scores using receiver operating characteristic (ROC) analysis.

Results

Of 58 patients, 55.2% had moderate to severe acute pancreatitis (AP) and 6.9% died. Alcohol was the leading etiology (63.8%) and was associated with all mortalities. Mean red cell distribution width-to-platelet ratio (RPR) was significantly higher in non-survivors (0.23) versus survivors (0.052). RPR showed excellent discrimination for mortality (Area Under Curve [AUC] = 0.986; cut-off ≥ 0.13) and severity (AUC = 0.932; cut-off ≥ 0.043) in patients of acute pancreatitis. Persistently elevated RPR correlated with prolonged ICU and hospital stay.

Conclusion

RPR is a simple, rapid, and cost-effective biomarker for predicting severity and mortality in acute pancreatitis, performing comparably to established scoring systems and suitable for early risk stratification in resource-limited settings.

## Introduction

Acute pancreatitis is one of the leading causes of hospitalization among gastrointestinal diseases. The underlying pathophysiology involves both localized and systemic inflammatory responses, which may progress to multi-organ failure. The disease course is variable: most patients present with mild, self-limiting disease and have a mortality rate of <1%, whereas approximately 20% may develop moderate to severe disease. Early mortality is typically due to systemic inflammatory response syndrome (SIRS)-related organ dysfunction, while late mortality is usually caused by infectious complications of pancreatic or peripancreatic collections. Given this variability, early differentiation between mild and severe forms of acute pancreatitis is crucial to guide management and resource utilization [1].

Although several clinical and laboratory predictors exist - including systemic inflammatory response syndrome (SIRS), C-reactive protein (CRP), creatinine, blood glucose, and hematocrit - these parameters alone are limited in accuracy. Complex multi-parameter scores such as Ranson's Criteria, Glasgow-Imrie Score, and Acute Physiology and Chronic Health Evaluation II (APACHE II) improve prediction but are cumbersome for routine bedside use [2]. Therefore, there remains a need for a simple, inexpensive, and universally available prognostic marker.

Complete blood count (CBC) is a widely available investigation that includes red cell distribution width (RDW), an index reflecting variation in erythrocyte size. RDW has emerged as a prognostic biomarker in multiple diseases including celiac disease, colorectal cancer, and acute myocardial infarction [3]. Elevated RDW is thought to reflect systemic inflammation and bone marrow dysfunction. Conversely, platelet counts are known to decrease in inflammatory states due to cytokine-induced bone marrow suppression and consumption in microthrombosis. Previous retrospective studies have suggested RPR may be useful for risk stratification in acute pancreatitis [4].

The ratio of RDW to platelet count, referred to as the RDW-to-Platelet Ratio (RPR), combines these two indices. RPR has been reported as a useful prognostic marker in liver fibrosis, cirrhosis, primary biliary cholangitis, and cardiovascular disease [5,6].

This study was undertaken to prospectively evaluate the diagnostic accuracy of RPR in predicting outcomes in acute pancreatitis and to compare its performance with validated clinical scoring systems.

## Materials and methods

This prospective observational study was conducted in the Department of General Surgery at R.L. Jalappa Hospital and Research Centre, Kolar, from June to August 2025. The primary objective was to assess the prognostic value of the red cell distribution width-to-platelet count ratio (RPR) in predicting the severity and outcomes of acute pancreatitis. Ethical clearance was obtained from the institutional ethics committee before commencing the study, and written informed consent was obtained from all participants.

A total of 58 adult patients diagnosed with acute pancreatitis during the study period were enrolled and followed from admission until discharge or death. For each participant, demographic details, clinical history, and etiological factors were recorded. Laboratory investigations were performed at admission (baseline), at 48 hours, and at 72 hours to obtain red cell distribution width (RDW) and platelet count values. The RPR was calculated using the formula:

RPR = RDW (%) / Platelet count (10⁹/L) [7].

In addition to RPR, conventional prognostic scoring systems including Ranson’s score [8], the Acute Physiology and Chronic Health Evaluation II (APACHE II) [9], and the Bedside Index for Severity in Acute Pancreatitis (BISAP) [10] were also computed for each patient to compare their prognostic accuracy against RPR. The clinical severity of acute pancreatitis was categorized according to the Revised Atlanta Classification into mild, moderately severe, and severe disease [11].

Organ failure was classified as transient if it resolved within 48 hours or persistent if it lasted beyond 48 hours. The duration of intensive care unit (ICU) stay, total hospital stay, and survival outcomes were recorded for all participants.

Patients aged over 18 years who were admitted with a diagnosis of acute pancreatitis and provided informed consent were included in the study. Patients with anemia (including iron deficiency, megaloblastic anemia, or anemia of chronic disease), hematological disorders such as thalassemia or hemolytic diseases, hematological malignancies, or those unwilling to participate were excluded from the study.

The sample size was determined based on the findings of Arora et al. [7], who reported a sensitivity of 89.17% for RPR in predicting severity at a cut-off value of 0.043. Using this sensitivity and applying the formula

$N = \frac{(Z_{\alpha/2})^2 \, P(1 - P)}{d^2}$

where P represents sensitivity (0.8917), Zα/2 corresponds to 1.96 for a 95% confidence level, and d is the desired precision (0.08), the estimated sample size was calculated to be 58 subjects. Accordingly, 58 patients with acute pancreatitis were included in this study.

Data entry was performed using Microsoft Excel (Microsoft Corp., Redmond, WA, USA), and statistical analysis was carried out with IBM SPSS Statistics for Windows, Version 26.0 (IBM Corp., Armonk, NY, USA). Continuous variables were expressed as mean ± standard deviation (SD), median, range, and interquartile range (IQR). Categorical variables were analyzed using the Chi-square test, and a p-value <0.05 was considered statistically significant. The prognostic accuracy of RPR and conventional scoring systems was evaluated using Receiver Operating Characteristic (ROC) curve analysis to determine the Area Under the Curve (AUC), optimal cut-off values (based on Youden’s index) [12], sensitivity, and specificity.

## Results

A total of 58 patients with acute pancreatitis were included in the study. Among them, 54 patients (93.1%) survived, while four (6.9%) were non-survivors. Based on severity, 26 patients (44.8%) had mild pancreatitis, whereas 32 patients (55.2%) had moderately severe or severe disease. Patients with moderately severe and severe pancreatitis developed organ dysfunction; however, organ dysfunction reversed within 48 hours in the moderately severe group, while it persisted in the severe group, leading to four deaths.

**Figure 1:**
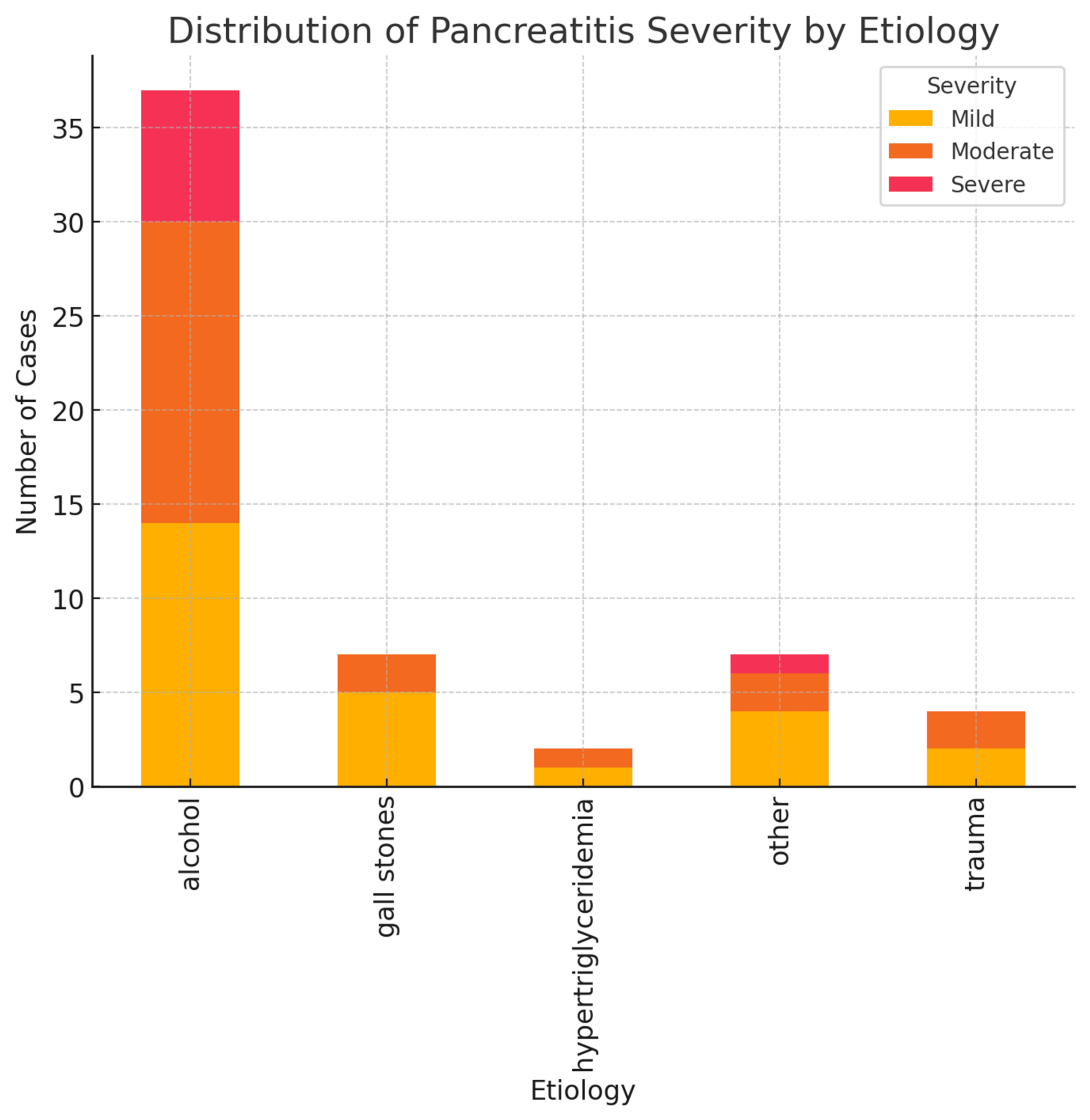
The distribution of etiology, severity, and mortality among patients with acute pancreatitis.

Etiology and Outcomes - Alcohol was the most common etiological factor, accounting for 63.8% (n = 37) of cases, followed by gallstones (12.1%, n = 7). Other causes included post-ERCP pancreatitis, autoimmune pancreatitis, and hypercalcemia (12.1%, n = 7), while trauma and hypertriglyceridemia contributed to 6.9% (n = 4) and 3.4% (n = 2) of cases, respectively. The distribution of etiology, disease severity, and mortality is summarized in Table 1. Alcohol was the only etiology associated with mortality, with four deaths, corresponding to a 10.8% mortality rate. The same findings are illustrated graphically in Figure 1, which depicts the relationship between etiology, disease severity, and mortality among patients with acute pancreatitis.

**Table 1:** The distribution of etiology, severity, and mortality among patients with acute pancreatitis.

Etiology	Mild	Moderate	Severe	Total	Deaths	Mortality %
Alcohol	14	16	7	37	4	10.8%
Gallstones	5	2	0	7	0	0%
Hypertriglyceridemia	1	1	0	2	0	0%
Other	4	2	1	7	0	0%
Trauma	2	2	0	4	0	0%

Baseline RPR Characteristics - The baseline characteristics of the red cell distribution width-to-platelet ratio (RPR) are presented in Table 2.

**Table 2:** Baseline RPR Characteristics

Statistic	Value
Number of patients (n)	58
Mean RPR	0.064
Median RPR	0.044
Standard Deviation (SD)	~0.065
Range	0.025 – 0.42
Interquartile range (IQR)	~0.029 – 0.067

One patient left against medical advice following baseline RPR measurement and was excluded from further analysis, resulting in a final study cohort of 57 patients.

**Figure 2:**
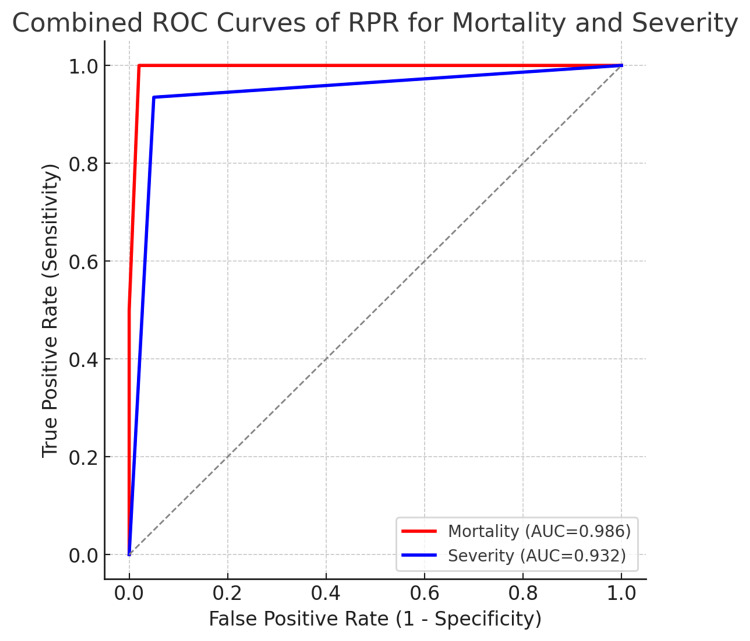
The ROC curve of RPR for both mortality and severity in patients with acute pancreatitis.

RDW-to-Platelet Ratio (RPR) and Mortality - Non-survivors had a mean RPR of 0.23 and a median of 0.185, whereas survivors had a mean RPR of 0.052 and a median of 0.044. Receiver Operating Characteristic (ROC) curve analysis revealed an area under the curve (AUC) of 0.986 for RPR in predicting mortality, with an optimal cut-off value of ≥0.13 as shown in Figure 2.

RDW-to-Platelet Ratio (RPR) and Disease Severity - When comparing disease severity, patients with mild pancreatitis had a mean RPR of 0.044 and a median of 0.032, whereas those with moderate or severe disease had a mean RPR of 0.082 and a median of 0.058. ROC analysis demonstrated that RPR effectively distinguished mild from moderate/severe cases, with an AUC of 0.932 at a cut-off of ≥0.043, as shown in Figure 2.

At this threshold, RPR achieved a sensitivity of 93.5% and specificity of 88.5%. Chi-square analysis further confirmed a strong correlation between high RPR values and the development of moderate to severe pancreatitis. Importantly, this cut-off value is lower than that used for mortality prediction, highlighting RPR as a continuum marker that increases progressively with both morbidity and mortality risk. These associations are summarized in Table 3 and Table 4.

**Table 3:** The relationship between RPR (≥0.13) and survival outcomes (n=57). Chi-square (χ²) = 45.8, Degrees of freedom (df) = 1 Chi-square p < 0.00000001, Fisher’s exact p = 1.27 × 10⁻⁵ High RPR (≥0.13) is very strongly associated with mortality.

RPR group	Non-survivors	Survivors
High (≥0.13)	4	1
Low (<0.13)	0	52

**Table 4:** The association between RPR (≥0.043) and disease severity. Chi-square (χ²) = 41.7, Degrees of freedom (df) = 1 Chi-square p < 0.00000001, Fisher’s exact p = 2 × 10⁻¹⁰ High RPR (≥0.043) is very strongly associated with moderate/severe disease.

RPR group	Mild	Moderate / severe
High (≥0.04)	3	29
Low (<0.043)	23	2

**Figure 3:**
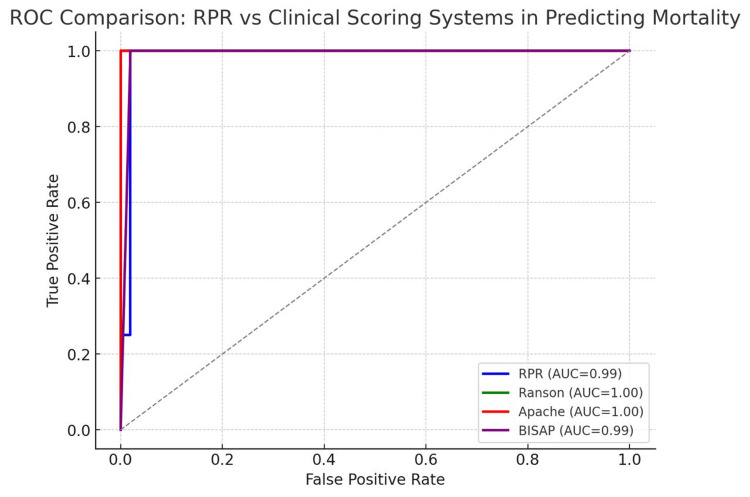
(Combined ROC curves) shows RPR with Ranson, APACHE II, and BISAP for mortality prediction.

Comparison with Conventional Prognostic Scores - The performance of RPR was compared with conventional prognostic scoring systems, including Ranson’s, APACHE II, and BISAP scores. As shown in Table 5, RPR exhibited excellent correlation with these established scores, achieving an AUC of 0.986 for predicting mortality, comparable to Ranson (AUC 1.000), APACHE II (AUC 1.000), and BISAP (AUC 0.990). The combined ROC curves of RPR and these scoring systems are illustrated in Figure 3, emphasizing the strong predictive ability of RPR for mortality in acute pancreatitis [7-10].

**Table 5:** Prognostic accuracy of RPR and conventional scoring systems.

Score	AUC	Best Cut-off
RPR [7]	0.986	0.13
Ranson [8]	1.000	4
APACHE II [9]	1.000	30
BISAP [10]	0.990	4

**Figure 4:**
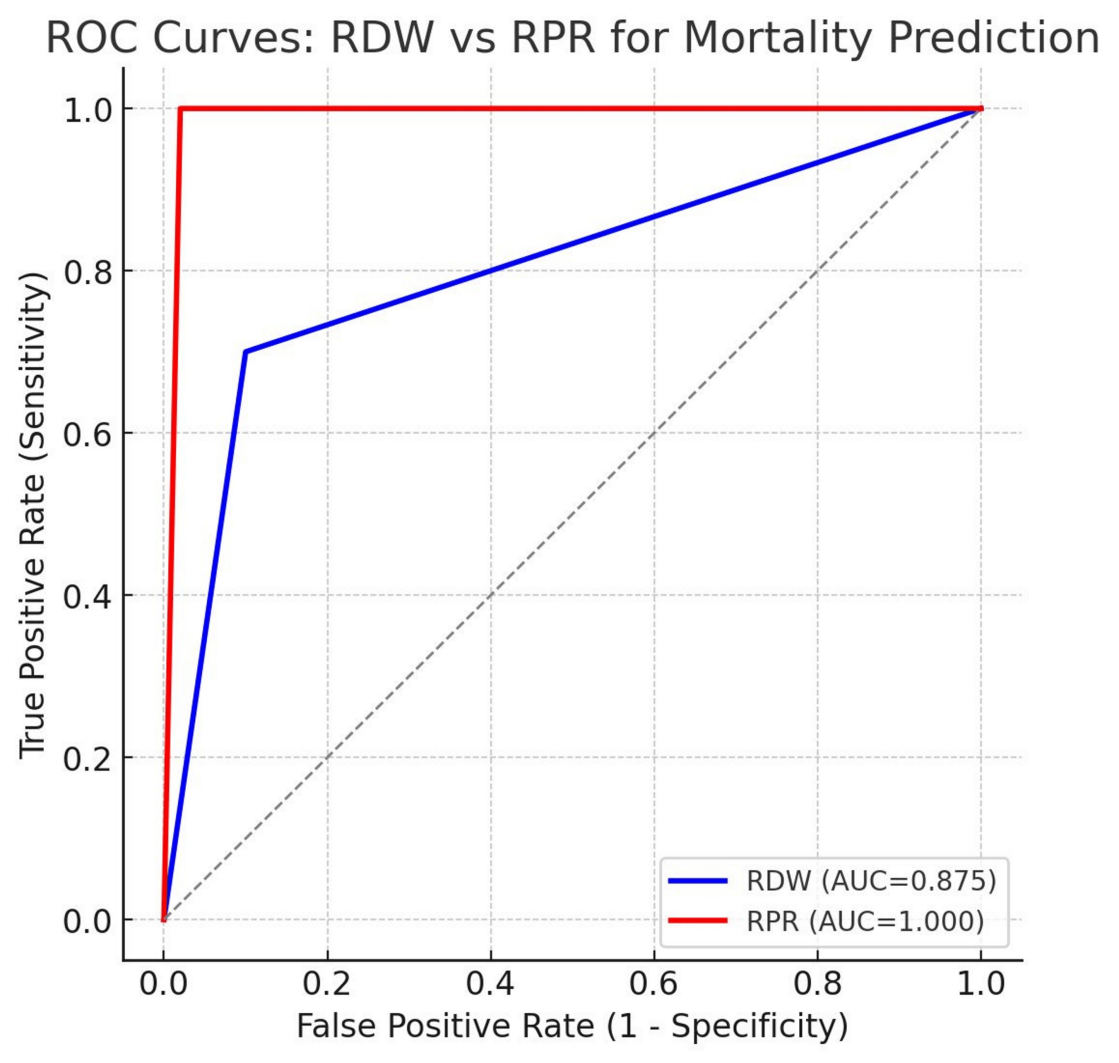
Compares ROC curves showing RPR outperform RDW in predicting mortality.

RDW vs RPR in Mortality and Morbidity - A comparative analysis between RDW and RPR revealed that RPR had superior prognostic performance. ROC analysis demonstrated an AUC of 0.875 for RDW and an AUC of 1.000 for RPR, indicating perfect mortality prediction in this cohort. Both RDW and RPR were higher in non-survivors compared to survivors. The detailed comparison is provided in Table 6, and the corresponding ROC curves are illustrated in Figure 4, which clearly shows RPR outperforming RDW in predicting mortality.

**Table 6:** Compares the prognostic performance of RDW and RPR in predicting mortality and morbidity. Adapted from Arora et al. [7].

Measure	RDW	RPR
AUC (Mortality Prediction)	0.875	1.000
Mean in Survivors	15.41	0.062
Mean in Non-survivors	18.58	0.230
ICU Stay (mean survivors)	4.5 d	4.5 d
ICU Stay (mean non-survivors)	2.0 d	2.0 d

**Figure 5:**
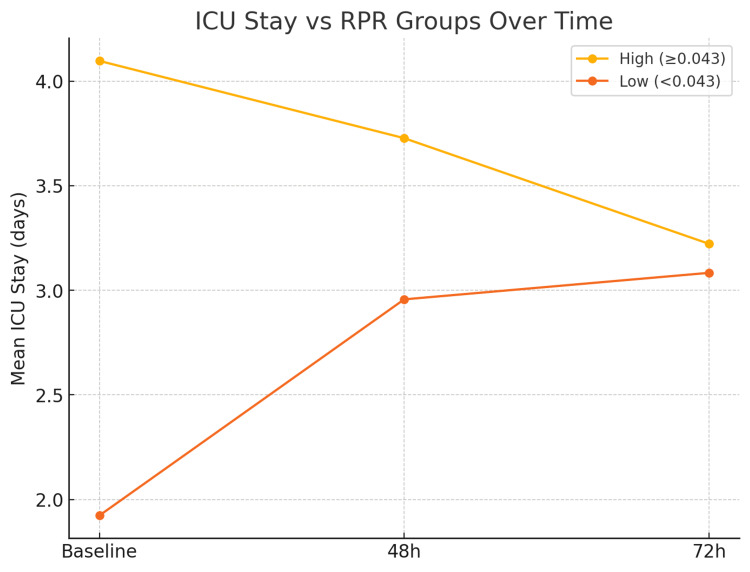
Demonstrates that persistently high RPR values are associated with prolonged ICU and hospital stays, whereas patients with low or falling RPR values had faster recovery.

Serial RPR and Its Association with ICU and Hospital Stay - Patients with high RPR values (≥0.043) consistently required longer ICU and hospital stays compared to those with lower RPR values. Although RPR levels declined slightly over time, persistently elevated values were strongly associated with prolonged morbidity. These findings suggest that serial RPR measurement can serve as a dynamic tool for ongoing risk stratification and disease monitoring. The relationship between serial RPR values and duration of ICU and hospital stay is presented in Table 7 and visually represented in Figure 5.

**Table 7:** Displays the mean ICU and hospital stay durations according to RPR group across different time points.

Timepoint	Group	ICU Stay (days)	Hosp. Stay (days)
Baseline	High (≥0.043)	4.1	12.9
	Low (<0.043)	1.9	9.5
48h	High (≥0.043)	3.7	12.6
	Low (<0.043)	3.0	11.1
72h	High (≥0.043)	3.2	11.9

## Discussion

The present study demonstrates that both red cell distribution width (RDW) and the RDW-to-platelet ratio (RPR) are valuable prognostic biomarkers in acute pancreatitis, with RPR consistently outperforming RDW in predicting adverse outcomes. Elevated RPR was strongly associated with both mortality and disease severity, with excellent discriminatory ability (AUC 0.986 for mortality and 0.932 for severity). These findings highlight the potential of RPR as a simple, inexpensive, and widely accessible tool for early risk stratification.

An additional strength of this study is the evaluation of serial RPR measurements. Persistently elevated RPR values were associated with prolonged ICU and hospital stays, suggesting that RPR may also serve as a dynamic marker to monitor disease trajectory. This supports its potential role not only in admission risk stratification but also in guiding ongoing clinical decision-making.

When compared with established prognostic scores (Ranson, APACHE II, BISAP) [8-10], RPR performed nearly as well, with AUC values approaching those of conventional models. Unlike these complex scores, which require multiple clinical and biochemical parameters, RPR can be derived instantly from a routine complete blood count. This makes it particularly attractive for use in resource-limited settings or emergency care, where rapid decision-making is critical.

Interestingly, we observed that non-survivors had shorter ICU stays compared to survivors. This paradoxical finding likely reflects early mortality, where rapid deterioration limited ICU duration. In contrast, survivors often required prolonged monitoring and supportive care, leading to longer ICU admissions. This underscores the importance of interpreting ICU stay cautiously, as it may reflect both disease severity and timing of death.

In contrast to Singh et al. (2020), who reported a male predominance of 69% with gallstones as the leading etiology (64%), our cohort showed a higher male predominance (~80%) with alcohol being the most common cause (63.8%).

Singh et al. also found a mean RDW of 14.1 ± 3.12 at admission with an AUC of 0.894 for predicting severity, whereas in our study, RDW yielded an AUC of 0.875, and the RDW-to-platelet ratio (RPR) outperformed RDW with an AUC of 0.986 for mortality prediction, making the ratio more susceptible indicator [13].

An imbalance between the early SIRS and the later compensatory counter-inflammatory response (CARS), and development of multiorgan failure are considered to be the primary causes of morbidity and mortality in severe acute pancreatitis [14].

Similar to the study of Zhang et al., our study also depicts that RDW is greater in the non-survivors than in the survivors [15].

In contrast to Vo et al. (2023), who reported RDW as a fair predictor of severity (AUC 0.73, cut-off 13.1%, Se 76.2%, Sp 69.1%), our study demonstrated a much higher discriminative capacity of both RDW (AUC 0.935) and especially RDW-to-platelet ratio (RPR; AUC 0.986) for predicting mortality in acute pancreatitis [16]. But RPR benefits over RDW with higher sensitivity and specificity.

Findings corroborate those of Çetinkaya et al. (2014) [17], who described RPR as an independent predictor of mortality in acute pancreatitis (AUC 0.783, cut-off 0.000067). However, in our cohort, RPR demonstrated markedly superior discriminatory ability (AUC 0.986 for mortality, cut-off 0.13; sensitivity 100%, specificity 98.1%), and uniquely correlated with morbidity indices such as ICU and hospital stay.

In severe acute pancreatitis, RDW rises due to systemic inflammation, cytokine surge, and bone marrow dysfunction, while platelet counts often fall from consumption and cytokine-mediated suppression; these changes reflect disease severity and predict adverse outcomes. The combination of these two parameters into RPR therefore provides a more robust reflection of the systemic inflammatory burden in acute pancreatitis.

RDW was shown to outperform conventional scoring systems for mortality prediction. The combination of RDW and platelets into the RPR further enhances prognostic accuracy, offering a simple, inexpensive bedside tool [17].

Hu et al. showed that increased RDW and low platelet count on admission in adults are associated with unfavorable outcomes on the short and long-term, similar to our study, which showed high RPR having more association with mortality [18].

RDW to platelets ratio has shown to have similar predicting value in mortality associated with other conditions like myocardial infarction [19] and heart failure [20].

Limitations

This study has certain limitations. Being a single-center study, the findings may not fully represent broader demographic or clinical variations. The relatively small number of deaths (n = 4) limits the statistical strength for mortality prediction and may reduce the precision of the estimated associations. Furthermore, the study lacks external validation in larger and more diverse populations, which is essential to confirm the reliability of the proposed RPR cut-off values and to assess the utility of incorporating RPR into existing prognostic models. Future multicentric studies with larger cohorts are therefore needed to strengthen and generalize these observations.

## Conclusions

The present study demonstrates that the red cell distribution width-to-platelet ratio (RPR) is a powerful, easily obtainable, and cost-effective biomarker for predicting outcomes in acute pancreatitis. Elevated RPR at admission was strongly associated with mortality and severe disease, with predictive performance comparable to established scoring systems. Furthermore, serial RPR measurements correlated with ICU requirement and prolonged hospital stay, underscoring its role as a marker of morbidity. Unlike complex prognostic scores that require multiple clinical and laboratory inputs, RPR can be calculated immediately from a routine complete blood count, making it highly practical for early risk stratification, especially in resource-limited settings.
